# Asymmetry of Muscle Mass Distribution and Grip Strength in Professional Handball Players

**DOI:** 10.3390/ijerph18041913

**Published:** 2021-02-16

**Authors:** Marcin Lijewski, Anna Burdukiewicz, Jadwiga Pietraszewska, Justyna Andrzejewska, Aleksandra Stachoń

**Affiliations:** Departament of Physical Anthropology, University School of Physical Education, 51-612 Wrocław, Poland; lijek22@gmail.com (M.L.); jadwiga.pietraszewska@awf.wroc.pl (J.P.); justyna.andrzejewska@awf.wroc.pl (J.A.); aleksandra.stachon@awf.wroc.pl (A.S.)

**Keywords:** bilateral asymmetry, muscle mass, hand grip strength, handedness, handball

## Abstract

Handball is among the disciplines that impose a significant degree of asymmetry on the body movement. The aim of the study is to assess the influence of physical effort on the occurrence of asymmetry in body musculature and in isometric strength of handball players. The study examined 36 professional handball players. Players’ height and body mass were measured as to calculate their body mass indexes (BMIs). Segmental bioelectrical impedance analysis (SBIA) was used to assess: the percentage of fat mass, total muscle mass (MM), musculature of the right and left side of the body, and body segments (trunk, upper and lower limbs). Moreover, grip strength was also measured. The assessment confirmed the existence of discrepancies in the right and left sides of players’ bodies for the majority of the parameters. Cross-asymmetry and significant bilateral discrepancies in trunk musculature were also observed. Morphological asymmetry may impact performance in sports since it can cause unfavorable functional changes, which in turn increase the risk of injury and conditions caused by overexertion. Therefore, we believe it is important to emphasize the importance of individualized symmetrization during sports practice and consistent monitoring of the asymmetries occurring in different body parts; this should both improve one’s sports results and minimize the risk of injury.

## 1. Introduction

Humans are characterized by bilateral symmetry which is set off by the plane that runs alongside the longitudinal axis of the body and divides the body into the left and the right part. Unlike the other primates, in humans, the functional preference for one of the upper limbs establishes directional asymmetry [1,2], which means that bilateral discrepancies occur consistently across the population. Right upper limb is functionally dominant in most humans. It is estimated that the frequency of left-handedness equals 10–13% [3]. The research has shown that left-handed sportsmen tend to be overrepresented on the professional level in the contact sports, whereas no such dependence has been found in the non-contact sports [4]. This phenomenon can be explained by the fact that the functional preference for the left upper limb provides an advantage in one-on-one combat, as it leads to behaviors that are difficult to anticipate and can thus directly improve one’s results.

Behavioral lateralization in humans manifests itself as directional asymmetry of the morphological features; the extent to which the asymmetry occurs is dictated by the differences in mechanical exertions that lead to deviations from perfect symmetry [5]. In humans, functional preference for the right upper limb causes its pronounced domination over the left upper limb in terms of the morphological features. Regular sports practice may also cause asymmetrical development of bones and musculature of the dominant limb [6]. The research conducted with the dual-energy X-ray absorptiometry (DXA) method has shown that even 12-weeks of practice can impact muscle and bone volume [7].

Other research has demonstrated that the preference for a particular upper limb increases that limb’s size. This is particularly visible in the hand’s width measurements and the hand’s length to width rate. The ambidextrous individuals have also shown hand asymmetry akin to that in left-handed individuals [8]. Moreover, upper and lower limbs exhibit cross-asymmetry that is caused by the asymmetrical use of the upper limbs, which in turn contributes to the asymmetry of the lower limbs in the direction opposite to that of the upper limbs [5]. The models that describe the impact of right-handedness on the lower body also show a significant degree of morphological discrepancies within the pelvis, indicating the domination of the left side of the lower body [9]. Auerbach and Ruff (2006) [10] have also shown that, within the lower limbs, directional asymmetry is less marked in the measurements of their length; yet, it is visibly marked in terms of the width of diaphysis and epiphysis of the long bones.

The degree of asymmetry is determined by the genetic and environmental (especially mechanical) factors; to some extent, it is also influenced by one’s lifestyle (physical labor and physical activity). Any exertion caused by sports practice may cause discrepancies in the formation of morphological features across the two sides of the body. The research conducted on young tennis players has also shown that while it grows, human skeleton is highly adaptable to any mechanical exertion or burden [11]. Regular sports practice of the young players resulted in certain changes in the dominant arm: an increase in bone size and fat-free mass, and, to a lesser extent, also in better mineralization. Moreover, the humeral asymmetry in younger players was similar to that in the professional tennis players; this may indicate that the adaptation of bone size caused by tennis may occur before or during adolescence. Similar results were obtained by Ducher et. al. (2005) [12], who used state-of-the-art approaches that allow for precise assessment of the asymmetrical changes that occur in particular tissues. The examination of the distal radial epiphysis of the dominant limb in tennis players with the use of magnetic resonance imaging (MRI) and dual-energy X-ray absorptiometry (DXA) has indicated the increase in bone size and bone mineral content, which influences bone strength. However, no correlation was found between the asymmetry in bone geometry and muscle volume. Based on these findings, it can be inferred that the physical activity initiated in childhood improved the mechanical capacity of the exerted bones through the changes in their length and height.

In sports, the side-to-side discrepancies in body morphology may also depend on the characteristics of the particular discipline that the given sportsmen practice [13]. An analysis was conducted on the influence of the types of martial art techniques and focused physical effort on the occurrence of asymmetry in body musculature and isometric strength in bodybuilders and in competitors of selected martial arts; its results indicated increased directional asymmetry in judo competitors. Less pronounced bilateral differences in the strength of musculature have been found in jiu-jitsu competitors and bodybuilders, whereas the participants who did not practice any sports displayed more pronounced cross asymmetry. Similarly, the asymmetrical nature of tennis causes uneven distribution of muscle mass [14]. The researchers have discovered significant differences between the dominant and non-dominant upper limbs in tennis players. The players showed a larger muscle mass of their dominant upper limbs. Moreover, no significant correlation was found between the age of the sportsmen or the length of their sports practice experience and the asymmetry coefficient. In athletes participating in 100-m runs, a trend to perform better was found in the athletes with more symmetrical structure of their knee joints and ankle joints [15]. An analysis of bilateral differences in javelin throwers has indicated that the asymmetry occurs in the length of shoulder and arm, in upper limb circumference, and in skinfold thickness [16]. Similarly, male modern pentathletes examined with the use of segmental analysis have shown unequal distribution of the muscle mass, which was particularly marked in the upper limbs and less marked in the lower limbs. In the female pentathletes, no statistically significant differences in the muscle mass have been found [17].

The differences in the muscle distribution were also found in academic players who practiced ball sports (baseball, soccer, tennis, and lacrosse). The lacrosse, baseball and tennis competitors were characterized by the overdevelopment of the forearm muscles, due to the nature of those sports. The soccer players have shown greater development of the musculature of the lower limbs and a more significant asymmetry between the dominant and non-dominant lower limb [18]. The substantial increase in muscle mass and in bone mineral density on the left side of the body has also been found in professional field hockey players, both male and female [19,20].

Both anthropometric and body composition measurements of soccer players from nine age categories have shown that, as their age increased, a decrease would occur in their fat mass, alongside an increase in the fat-free body mass and the side-to-side differences in the musculature of the lower limbs. In junior competitors, no morphological asymmetry of the lower limbs has been found [21]. Moreover, research has found that in football players, long-term routine physical exertion can influence the morphological structure of their lower limbs and, as a result, the expansion of the bone mass, the bone cross-sectional area, and the thickness of the cortical layer of the tibiae [22]. It has also been shown that the more efficient marksmen were also characterized by the lesser degree of asymmetry in the lean mass and the strength of their lower limbs. A connection has also been found between the accuracy of marksmen and the increase in their lean mass, as well as the decrease in their fat mass [23]. Conversely, in the elite futsal players, no bilateral asymmetries (be it morphological, functional, or neuromuscular) asymmetries have been found in the lower limbs. The less successful players were also characterized by significant discrepancies in fat mass and lean mass between the dominant/non-dominant limbs. It is the consequence of the fact that futsal is less dynamic in contrast with soccer and is also played on a far smaller court. Examination of academic sportsmen has also allowed the researchers to inspect the impact of the degree of bilateral differences in the lean body mass of the lower limb on the asymmetry of strength and power when jumping [24].

As shown in the research review presented herein, competitors from various sports disciplines exhibit discrepancies in the degree of body asymmetry that are caused to one-sided exertion. Team sports, such as handball, volleyball, and basketball, have not yet been sufficiently described in academic studies that examine the involvement of a particular side of the body over the other. Handball is characterized by a significant asymmetry in body movement; therefore, it is reasonable to hypothesize that marked two-sided morphological discrepancies will develop in the players who use one side of the body more frequently than the other during their practice and matches. The aim of the study was to assess the influence of focused physical effort on the occurrence of asymmetry in body musculature and in isometric strength of handball players.

## 2. Materials and Methods

The study examined 36 professional handball players aged 26.1 ± 6.44. The players had prior experience of 14.4 ± 6.89 years in handball. Among them, 9 players declared that they use their left upper limb not only in activities requiring precision but also when playing handball (throwing, passing, etc.). That number of left-handed players comprises 25% of the group and amounts to a number that is higher than the percentage of left-handed people in the overall population (7–11.8%) [25].

The measurements were taken at the end of the preparation period, before the start of the competitive season. The study was approved by the Ethics Committee of the University School of Physical Education in Wrocław, Poland (2/2020), and was conducted in accordance with the requirements stipulated in the Declaration of Helsinki. Participants were fully informed about all experimental procedures, and a written informed consent was obtained from all of them. A survey was used to collect information regarding the participants’ date of birth, length of their sports practice experience, supplements taken, and any physical traumas they might have experienced. Athletes previously reported that they did not use any exogenous anabolic androgenic steroids, drugs, medication, or dietary supplements.

The measurements were taken during the morning hours. The measurements were conducted by two experienced anthropometrists in accordance with the anthropometric measurement standards delineated by International Society for the Advancement of Kinanthropometry (ISAK). Body height was measured with the use of an anthropometer accurate to up to 0.1 cm (GPM Siber Hegner Machinery Ltd., Zurich, Switzerland); the body mass was measured with the use of an electronic scale accurate up to 0.1 kg (Fawag, Lublin, Poland). The somatic features were used to assess the weight-height proportions. In order to do that, BMI was calculated (body mass (kg)/body height (m)^2^).

Moreover, fat mass and muscle mass (MM) distribution were estimated with the use of segmental bioelectrical impedance analysis (SBIA). Bioelectrical impedance analysis (BIA) is an inexpensive and noninvasive method for assessing body composition [26]. Moreover, it is a portable, fast, and easy to use method for body composition assessment in field research [27]. Segmental BIA (SBIA) makes it possible to assess both the entire body composition and [28] the musculature of its segments (lower and upper limb, trunk). Throughout the measurements, standardized conditions for a bio-impedance measurement were maintained [29].

An analyzer with a built-in BodyScan module was used: BIA-101 Anniversary Sport Edition made by Akern (tetrapolar and octopolar version, electrode position: Hand–foot, BodyGram 1.31 software, BodyScan 5.0; Florence, Italy). Body composition measurements were taken in accordance with manufacturer’s recommendations (the subjects were in fasting state during the measurements, they were lying on their backs in a horizontal position, their limbs were resting at 40 degree angle from the body and the time between the measurement and their last physical strain was 12 h or more). The analysis took the following elements of body composition into account: percentage fat mass, complete muscle mass (kg), the muscle mass of the left and right body sides and the muscle mass of the body segments (trunk, upper and lower limb) on the right and left side. The muscle mass on the right side of the trunk was calculated by subtracting the total muscle mass on the right lower and upper limbs from the muscle mass on the right side of the body. The muscle mass on the left side of that body segment was calculated using the analogous method. The components of the body composition are expressed in absolute values (kg).

Moreover, grip strength was also measured. Grip strength plays an important role in throwing and casting the ball and other equipment across various sports disciplines. This physiological variable is influenced by a multitude of factors, such as age, gender, and body size [30]. Grip strength was measured with the use of the hand grip dynamometer (T.K.K.5001, Takei Scientific Inst. Co., Ltd., Niigata, Japan). The aim of this test was to measure the maximum isometric strength of the muscles in palm and forearm. During the measurement, the straightened upper limb was directed towards the ground [31]. The players performed 2 repetitions at maximum intensity with a three-minute rest between trials to minimize the effects of fatigue.

### Statistical Methods

The tests were carried out with the use of Statistica 13 package (Dell Inc., Tulsa, OK, USA). Shapiro-Wilk test was used to examine the distributions in the analyzed characteristics. The paired t-test was utilized to determine if right and left sides were significantly different for each dimension. Alpha level was set at *p* < 0.05.

The Standardized directional asymmetry (DA) and Standardized absolute asymmetry (AA) were also calculated. DA score is the quantitative measure of directional asymmetry in the muscle mass of particular body segments and grip strengths. It is calculated as [10]:

DA = (R − L)/(1/2 (R + L)) × 100%

where L = left measurement, and R = right measurement. The scores above zero mean that the right side measurements are higher, whereas the scores below zero mean that the left-side measurements are higher. Standardized absolute asymmetry (AA) score is the measure that does not account for the directionality of the asymmetry and is calculated as follows [10]:

AA = (|R − L|)/(1/2(R + L)) × 100%

Since the number of the left-handed competitors was small, normalized values (Z) were calculated for the mean and the standard deviation of the entire sample in order to examine the variability of the analyzed characteristics in left- and right-handed competitors.

Once the structure of the variables taken into account in the research was established, it was possible to apply cluster analysis. The distances in cluster analysis were calculated with the use of 1-r Pearson’s formula. Grouping was done with the use of Ward’s method [32], which is a hierarchical method of agglomerative analysis. A dendrogram is a graphical representation of structure within a set of characteristics. It groups the data points that differ the least from one another (have minimal variance within the clusters) and are connected on a given level of similarity. A dendrogram branches out based on the analysis of the consequences of dividing the taxonomic pyramid on different levels. The dendrogram’s branches should be set at the height that precedes a significant decrease of similarity between the sets of objects or clusters.

## 3. Results

Table 1 presents the statistical characteristics of the analyzed variables. Shapiro-Wilk test did not indicate any departure from the normal distribution of the analyzed characteristics.

Muscle mass of the right side of the body is significantly higher compared to that of the left side (Table 2). A similar relationship can be observed in the muscle mass on the two sides of the trunk. Bilateral variability in the grip strength of the hands is statistically significant, as well. No statistically significant differences were found in the muscle mass of the right and left lower and upper limbs. The lack of a clear differentiation of the upper limb musculature can be partially explained by the presence of left-handed individuals in the studied group of players.

**Figure 1:**
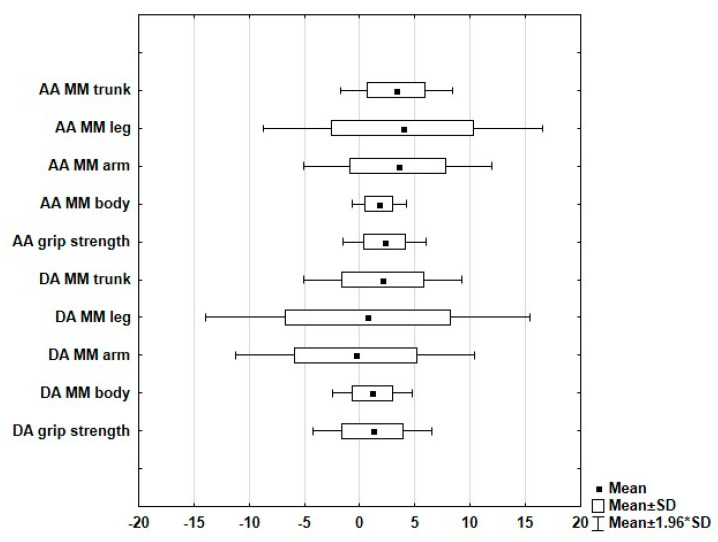
Mean standardized directional asymmetry score (DA) and mean standardized absolute asymmetry score (AA) for the analyzed characteristics of the musculature (MM-muscle mass).

Analysis of bilateral variability in the muscle mass has shown that in the case of the upper limb musculature, the directional asymmetry (DA) index is negative, which indicates that there is less musculature on the left side (Figure 1). Positive values for DA were obtained for the musculature of the trunk, total body and the lower limbs. Largest variability can be found in the differences between the muscle mass values of the lower and upper limbs. DA index for grip strength indicates the prevalence of the right hand. The absolute asymmetry (AA) indices that ignore the directionality of the asymmetry are the highest for the upper and lower limbs. Side-based variability of trunk muscle mass is higher in comparison with the analogous value for the total body.

**Figure 2:**
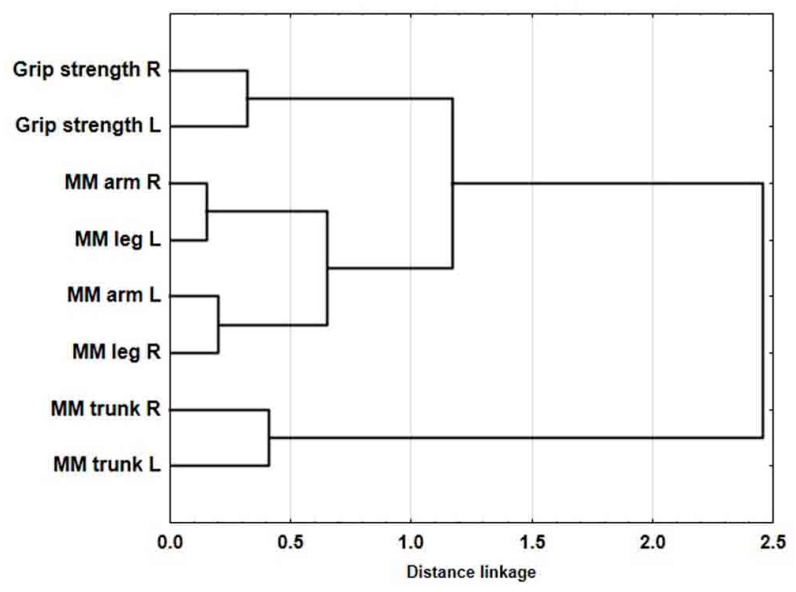
Clusters of the somatic and functional characteristics of the handball players.

The agglomeration method in relation to the selected variables revealed four clusters (Figure 2). Low agglomerative coefficients are assigned to the two-element clusters that represent the musculature of the right upper limb and the left lower limb and the left upper limb and the right lower limb. They represent cross-asymmetry. These measurements of muscle strength comprise a separate cluster. The cluster of the characteristics that represent the musculature of the right and left side of the trunk has the weakest relationship with the above mentioned clusters.

**Figure 3:**
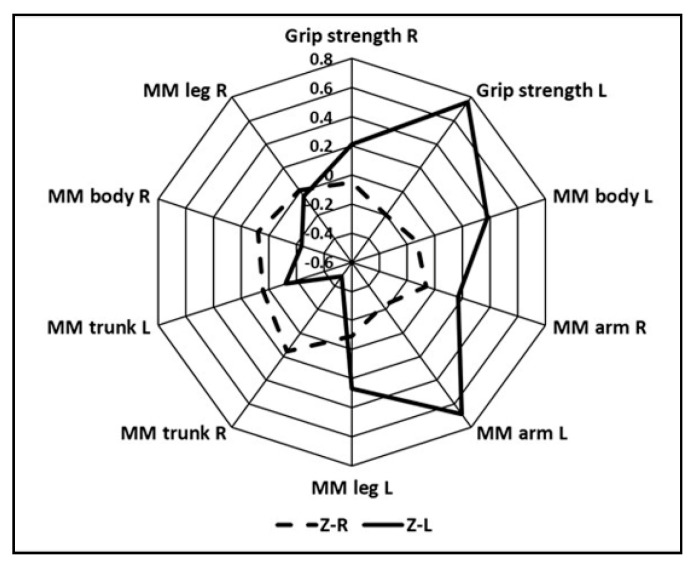
Normalized values (Z-score) of the musculature characteristics and grip strength of the hands of the right-handed players (Z-R) and the left-handed players (Z-L).

Since the number of the left-handed competitors was small, variability across the groups of the analyzed variables was assessed through normalizing the arithmetic mean and standard deviation of the entire sample (Figure 3). It was noted that the left-handed competitors are characterized by a far higher grip strength of both hands. The difference is higher in the case of the left hand. Moreover, the muscle mass of the left side of the body, left lower limb, and both upper limbs is higher in the left-handed players. Right-handed players have significantly higher muscle mass for their trunk and right side of the body. However, the muscle mass of their lower right limb is similar in both groups.

## 4. Discussion

Our research analyzed the body composition of the professional handball players and the influence of sports practice on the grip strength and the asymmetry of body musculature. We used cluster analysis to examine the morpho-functional structure. We also examined the variability in the musculature of the body and in its segments in the groups of players divided on the basis of their handedness.

In relation to the Polish population, the players examined exhibit the following characteristics: body height at 90th centile, body mass above 90th centile, and BMI above 85th centile [33]. Fat mass percentage amounted to 17.7% and is higher than that obtained by other research [34,35], which is the result of different measurement methods; our research uses BIA, whereas the other researchers used anthropometric measurements and DXA. Anthropometric measurement is cheap but requires far more time and effort, whereas DXA, though very precise and infallible, is very expensive to use and cannot be applied outside of the laboratory. The BIA method is quick and easy to use for assessing changes caused by sports practice, as shown in other research [36]. The other limitation of the study was measuring only grip strength as muscle performance, without lower limb strength.

In handball players, well-developed musculature is the foundation of their strength and power [24,37]. In people who do not practice any sports, the discrepancies in musculature between right and left sides of the body are small and confirm the general pattern caused by the bilateral asymmetry of the human body [38]. In handball players, however, statistically significant side-to-side differences of the muscle mass have been found for right and left sides of the body, as well as trunk, which is caused by the particular motor requirements imposed by handball. As shown by other research, ball velocity was significantly impacted by the run-up, as well as pelvis rotation and trunk movements [39,40]. Depending on the type of the move used—standing throw or a jump throw—, professional players would apply different techniques to accelerate the movement of their pelvis and trunk to gain extra ball speed: lead leg braces the body versus opposed leg movements during flight. These techniques require the use of the muscles that stabilize the pelvis (*glutues medius*) and the lower back (*quadratus lumborum*). Adductor muscles are also particularly important as they support the stabilization of the pelvis, ensure body balance, and take part in the movement of the lower limbs. Moreover, they make it possible to rotate and bend the hip joint, to bend the knees, and to straighten the hip.

Since the vast majority of handball moves use one leg, an uneven amount of strain is required by the two sides of the body; this influences the increase in the muscle mass of the lower right limb compared to the lower left limb that our research has found. The more developed musculature generates more power and strength, which are very important for player effectiveness. As shown by other research, handball throwing velocity is strongly associated with lower-limb strength, although upper-limb strength, jumping, and sprint capacities also play a relevant role in throwing performance [41]. The handball jump throw involves executing a vertical jump off one leg allowing the player to take off after the run-up, while the main part of the throwing movement is executed during the flight phase; therefore, the strength of the lower limbs is of particular importance for the execution of team and individual maneuvers during the game [42]. Moreover, previous research [24] has also shown that the asymmetry of the fat-free mass, including not only the muscle mass but also bone mass, may also explain the asymmetry of strength and power generated during the jump.

Our research has also indicated a greater muscle mass of the right upper limb and significantly higher grip strength. Since the particular technique is required from a handball player, a significant amount of burden falls on their dominant arm and shoulder, which means that this particular limb does also develop greater strength, resulting in its structural asymmetry [43,44]. The handball throw is characterized by large external shoulder rotation followed by a rapid internal rotation with minor changes in shoulder flexion and abduction. Throwing speed is the highest in the standing throw with run-up, slightly lower in the standing throw without run-up, lower for the jump throw, and the lowest for pivot throw [45].

The analysis of the structure within the set variables with the use of the agglomerative method has highlighted a number of discrepancies; these involve the development of musculature across the body segments, indicating very marked cross-asymmetry. Moreover, low agglomerative coefficients have been assigned to the two-element clusters that represent the musculature of a) the right upper limb and the left lower limb; and b) the left upper limb and the right lower limb. This is the result of the throwing technique discussed hereinabove and the effect of the tissue adaptation to the burden induced by physical effort. In handball players, unlike the general population, the absolute asymmetry (AA) indices that ignore the directionality of the asymmetry are the highest for the lower limbs, which indicates the high involvement of that body segment. The muscle mass in the right and left side of the trunk constitutes a separate cluster and shows the lowest connection to the musculature traits of the limbs, which indicates that this segment is separated. However, many electromyographic tests have shown that the spinal erector muscles are important core muscles that control the movement patterns during walking and other rhythmical motor tasks [46]; research has also shown that those muscles play a key role in sports [47].

We also examined the fluctuations in the musculature of the body and in its segments depending on players’ handedness. Human handedness is connected to a multitude of cognitive and behavioral properties, including hazardous behavior, which may explain why the proportion of the left-handed people is higher in sports than in the general population [48,49]. Within the examined sample, nine players declared that they preferred their left upper limb; they comprised 25% of the entire group and amounted to a number that was significantly higher than the percentage of left-handed people in the overall population (7–11.8%) [25,50]. This player distribution is also in line with the research; it is claimed that it is important for a team to have several left-handed players on the roster, especially in the “back” positions to improve the team’s effectiveness on the right side of the court [51]. Our research indicates that handedness influences the side-to-side discrepancies in the muscle mass of the body segments, as was also confirmed by other researchers [52].

Professional left-handed handball players display less pronounced cross-asymmetry. They are also characterized by greater musculature of the left side of the body, left lower limb, and both upper limbs, the left limb in particular, which is the result of functional lateralization. Moreover, they also display greater grip strength in their upper left limb. Moreover, their non-dominant upper limb is also stronger, which is indicated by the greater muscle mass [53]. Right-handed players are also characterized by far greater musculature of the trunk and right side of the body, which is the result of the functional preference of that limb. However, the muscle mass of their lower right limb is similar in both player groups, confirming the tendencies discussed in other research [24].

## 5. Conclusions

Handball influences the asymmetrical increase of hypertrophy of body musculature in professional sportsmen. Our research has confirmed the existence of differences in right and left sides of players’ bodies for the majority of the inspected parameters. We observed cross-asymmetry and significant bilateral discrepancies in trunk musculature. Morphological asymmetry may impact players’ performance in sports since it causes unfavorable functional changes, which in turn increase the risk of injury and conditions caused by overexertion, e.g., back pain [54,55]. Therefore, it is crucial to emphasize the importance of individualized symmetrization during sports practice and consistent monitoring of the asymmetries occurring in different body parts; this should both improve one’s sports results and potentially minimize the risk of injury.

## Figures and Tables

**Table 1:** Basic statistics for the measurements along with Shapiro-Wilk Normality Test results (SD—standard deviation).

Variable	Side	Mean	SD	Shapiro-Wilk Statistic	*p*-Value
Anthropometry					
Body height [m]	-	1.86	0.67	0.937	0.065
Body mass [kg]	-	89.27	11.47	0.972	0.489
BMI	-	25.64	2.49	0.953	0.133
% Fat	-	17.17	4.18	0.984	0.872
Muscle mass total [kg]	-	54.91	6.16	0.976	0.614
Muscle mass trunk [kg]	-	28.06	4.16	0.901	0.055
Muscle mass body [kg]	R	28.03	3.43	0.975	0.567
Muscle mass body [kg]	L	26.88	3.26	0.985	0.889
Muscle mass arm [kg]	R	4.13	1.17	0.953	0.134
Muscle mass arm [kg]	L	4.18	1.07	0.969	0.422
Muscle mass leg [kg]	R	9.47	2.48	0.937	0.068
Muscle mass leg [kg]	L	9.07	2.99	0.964	0.285
Muscle mass trunk [kg]	R	14.43	1.92	0.974	0.546
Muscle mass trunk [kg]	L	13.63	2.77	0.955	0.143
Strength					
Grip strength R [kg]	R	56.49	9.27	0.939	0.059
Grip strength L [kg]	L	54.68	8.91	0.949	0.119

**Table 2:** Bilateral variability in muscle mass and grip strength in the handball players (D—the sample mean difference, SD—standard deviation).

Variable	D	SD	*p*-Value
Muscle mass body R—L [kg]	1.15	2.60	0.012
Muscle mass arm R—L [kg]	−0.05	1.01	0.767
Muscle mass leg R—L [kg]	0.40	2.47	0.339
Muscle mass trunk R—L [kg]	0.80	2.30	0.044
Grip strength R—L [kg]	1.81	7.31	0.003
